# Generating porous metal surfaces as a mean to incorporate thymol-loaded nanoparticles

**DOI:** 10.1186/s11671-023-03854-0

**Published:** 2023-06-20

**Authors:** Chalom Zemmour, Sofya Zakharova, Ofra Benny

**Affiliations:** 1 Faculty of Medicine, School of Pharmacy, Institute for Drug Research, The Hebrew University of Jerusalem, 91120 Jerusalem, Israel; 2 Bezalel Academy of Arts and Design Jerusalem, Jerusalem, Israel

**Keywords:** Metal, Porosity, Slow-release, Polymers, Nanoparticles

## Abstract

Porous metals have gained interest in many fields such as biomedicine, electronics, and energy. Despite the many benefits that these structures may offer, one of the major challenges in utilizing porous metals is to incorporate active compounds, either small molecules or macromolecules, on these surfaces. Coatings that contain active molecules have previously been used for biomedical applications to enable the slow release of drugs, e.g., with drug-eluting cardiovascular stents. However, direct deposition of organic materials on metals by coatings is very difficult due to the challenge of obtaining uniform coatings, as well as issues related to layer adherence and mechanical stability. Our study describes an optimization of a production process of different porous metals, aluminum, gold, and titanium, using wet-etching. Pertinent physicochemical measurements were carried out to characterize the porous surfaces. Following the production of porous metal surface, a new methodology for incorporating active materials onto the metals by using mechanical entrapment of polymeric nanoparticles in metal pores was developed. To demonstrate our concept of active material incorporation, we produced an odor-releasing metal object with embedded particles loaded with thymol, an odoriferous molecule. Polymer particles were placed inside nanopores in a 3D-printed titanium ring. Chemical analysis, followed by smell tests, indicated that the smell intensity lasts significantly longer in the porous material containing the nanoparticles, compared with the free thymol.

**Supplementary Information:**

The online version contains supplementary material available at 10.1186/s11671-023-03854-0.

## Introduction

Porous metals are a class of materials with unique properties, including high surface area, high permeability, and tunable pore size and distribution [1]. These properties make them suitable for a wide range of applications, such as catalysis, filtration, energy storage, and biomedical engineering. Porous metal surfaces are increasingly gaining interest due to their superior properties compared with other porous materials such as polymers or ceramics in terms of their mechanical stability, oxidation resistance, lightweight, and additional physical properties, e.g. electrical, acoustic, thermal, and vibration properties [2, 3]. The high surface area of porous metals allows a higher number of chemical reactions to occur at active sites, making them appropriate for catalytic applications. In filtration applications such as gas separation and water purification, the tunable pore size and distribution make porous metals useful for separating different-sized particles. Porous metals also have potential in energy storage applications, such as batteries and supercapacitors [4]. In the biomedical field, porous metals are used for bone implants, as they can promote bone tissue regeneration and integration. Some examples of porous metals include titanium, nickel, and stainless-steel foams, which are widely used in industrial and biomedical applications [5]. Overall, the unique properties of porous metals make them versatile and promising materials for various fields.

The porosity of the surface may provide important advantages in the integration of active molecules; environmental factors such as temperature, light, or humidity can alter the stability of active molecules [3]. Depositing molecules inside pores may protect them and potentially improve stability and shelf life. The release rate of the active material can be controlled by several factors, including the size and shape of the pores, the surface chemistry of the porous material, and the interaction between the active compound and the porous material [6, 7]. Shifting from dense to porous material increases the effective surface area available for interactions between the active components and the matrix [8, 9].Therefore, it may serve as an important strategy for improving the stability, controlled release, efficacy of various porous devices.

There are various methods for producing porous metals, including powder metallurgy, foaming, chemical vapor deposition (CVD), electrodeposition, and chemical etching [2]. In powder metallurgy, a mixture of metal powder and sacrificing filler material is sintered and then removed to create pores. Foaming involves creating a metallic foam by mixing a molten metal with a blowing agent and cooling it rapidly. In CVD, metal atoms are deposited onto a substrate to create a porous structure and in electrodeposition, metal ions are reduced at the cathode to form a metal layer with controlled porosity [10, 11]. These methods hold significant advantages in controlling the pores’ size and shape and having the ability to produce complex shapes. However, all these processes required special equipment, the procedure is relatively slow, not compatible for large surfaces, and may have limited mechanical strength [2]. Alternatively, in chemical etching method, a metal piece is treated with an etchant solvent to remove material and create pores. In this technique large surface of metal can be processed using low-cost resources. However, it is limited to certain types of metals and requires careful control to achieve consistent results. Despite the attractiveness of this methodology a systematic study for diverse materials was lacking so far. It should be noted that many factors affect the production of porous metals that are differ between metals. The pore size, distribution, and morphology of the resulting porous metal can be influenced by a variety of factors including etching solvent type and concentration, etching time, temperature, metal type, surface preparation, and agitation [12].

Here, we attempt to examine the various factors that can influence the synthesis of porous metals composed of various materials (with nanoscale pores). For this purpose, we have used aluminum, gold, and titanium to test different conditions that can provide a precise control over the pore mesh size.

In addition, these different porous metals have been used to integrate odoriferous molecules. In contrast to the current approaches of incorporating active materials on metals via coatings, we introduced a method that utilizes the pores in the metal surfaces to immobilize slow-releasing nanoparticles directly using the mechanical entrapment of particles. Therefore, it was crucial to regulate the metals' pore sizes to effectively entrap the nanoparticles. By optimizing the production protocol, these adjustments were made. For the proof of concept, we focused on developing smell-carrying metals as a potential way of utilizing odor-releasing objects. We fabricated nanoparticles composed of poly(lactic-co-glycolic acid) copolymer (PLGA) that can encapsulate active compounds for their slow release. PLGA is known for its excellent biocompatibility, biodegradability, and good mechanical strength, making it an attractive material for immobilization inside porous surfaces [13, 14]. The volatile odoriferous small molecule, thymol, was selected as a model compound to be encapsulated in the PLGA nanoparticles. These nanoparticles were then deployed on the metal's porous surface. To date, only a few studies have focused on releasing fragrances and odor molecules in porous matrices. The main challenge has been the ability to control the release rate of volatile molecules on dry surfaces. For example, this issue was explored in the textile industry as a method to minimize the foul smells of clothes after their production [15].

Porous titanium was used as a substrate for carrying nanoparticles with a size of about 150 nm, and that were physically entrapped in the pores. The thymol release profile was determined over several days by measuring the fraction of thymol that was released from the titanium-nanoparticle samples when exposed to air. Our results indicated that when thymol was encapsulated in nanoparticles, almost 40% of its initial quantity was retained on the surface after 15 days, compared with free thymol dried on the metal surface, used as a control. To further evaluate the ability to generate smell-releasing metal surfaces, we conducted a blind smell test that compared porous metals that contained either free thymol or thymol loaded in nanoparticles. For this study, we used porous titanium samples (1 cm by 1 cm) covered with dried thymol-loaded nanoparticles, and the smell test was performed for 10 days. In this evaluation we found a statistically significant difference between the samples; the slow-release samples showed higher retention of smell compared with non-porous materials. Finally, a 3D-printed prototype of an odor-releasing titanium jewel ring was designed and produced, indicating that the process can be performed in macro-objects with a three-dimensional shape, and not only with flat ones, and that the etching process does not compromise the visual appearance of the object.

Taken together, we present a novel platform that can be used for volatile-releasing metal objects by directly incorporating slow-releasing nanoparticles in the metal pores. Different porous metals were produced following detailed controllable process, potentially supporting versatile applications of this technology. This innovative method could be further applied in various metals and applications.

## Materials and methods

### Metal samples preparation

Pure titanium and aluminum foils (99% purity, 25 × 25 × 0.25 mm^3^) were purchased from Alfa Aesar (MA, USA), and 9-karat yellow gold sheets (10 × 7 × 0.2 mm^3^) were purchased from Rashbel (Israel). To produce nanoporous structures, the titanium samples were dipped in 5, 7.5, or 10 M NaOH (Bio-Lab Ltd, Israel) aqueous solutions for 24 h or 48 h; the gold and aluminum samples were dipped in 15% or 35% HNO_3_ (Acros Organics, Belgium) aqueous solutions for different durations. The samples were washed with DDW for 1 min and dried under a nitrogen stream.

### Thymol-loaded PLGA particles preparation and deposition

Nanoparticles were prepared using an emulsification-evaporation method. The oily phase was formed by dissolving 100 mg of PLGA 50:50 lactic according to a glycolic acid ratio (Sigma-Aldrich, MA, USA) and 20 mg of thymol (MW = 150.22 g/mol; ThermoFisher, MA, USA) in 10 mL of acetonitrile (Bio-Lab Ltd., Israel) containing 0.01% Tween 80 (ThermoFisher, MA, USA). While being stirred, the aqueous phase of Solutol 0.1% (Glentham Life Sciences, UK) was added to the oily phase and stirred for 10 min using an overhead stirrer at 550 rpm. Next, the solution was transferred into a round bottom flask and connected to a rotary evaporator (Heidolph, Germany). After the solvent was entirely evaporated, the nanoparticles were centrifuged at 10,000 rpm for 5 min to remove impurities. A solution of 0.5 mg/mL thymol-loaded PLGA particles was deposited on porous titanium. mPEG-b-PLA micelles, prepared by a dialysis method as previously described [16], with a size of 30 nm, and silica nanoparticles, purchased from Nanocomposix (CA, USA), with a size of 50 nm, were deposited on porous aluminum and gold surfaces. The volume of suspended nanoparticles was 20 µL for a 1 cm by 1 cm piece of metal; evaporation was carried out overnight at room temperature.

### Physical and chemical characterization of metal samples and nanoparticles

The nanoparticles' mean size, range, and charge were measured using dynamic light scattering and Zetasizer (Zetasizer Nano ZSP, Malvern Instruments) at 25 °C. For scanning electron microscopy (SEM, Magellan 400L ThermoFisher, a former FEI Company, USA), the metal and particle samples were sputtered with a thin iridium layer. For transmission electron microscopy (TEM, JEM-1400 Plus, JEOL, Japan), the particle samples were initially stained with 2.5% uranyl acetate, and 5 µL of the particle suspension was placed on formvar/carbon-coated copper 200 mesh grids and mixed with 5 µL of NanoVan (Nanoprobes, NY, USA) for 10 s. After the extra stain agent was removed, the grids were dried and measured. A high-resolution SEM (Apreo 2S, ThermoFisher, a former FEI Company, USA) equipped with energy-dispersive X-ray spectroscopy (EDS) technology was utilized to analyze the samples' chemical elemental composition.

Microhardness indentation measurements were performed to assess the mechanical stability of the samples. Vickers hardness was measured using a 100-gf load and a 15-s loading time (Duramin-40, Struers, Denmark) on each metal before etching treatment, 24 h after, and 3 months after. To evaluate the metal surface energy, contact angles for 5 μL of DDW droplets were measured on each surface. The contact angles were measured after processing by ImageJ free software using a contact angle plugin [17]. For these measurements, aluminum samples were dipped in HNO_3_ 35% for 35 min, gold samples were dipped in HNO_3_ 35% for 72 h and titanium samples were obtained were dipped in NaOH 5 M for 24 h after which they were washed with DDW for 1 min and dried under a nitrogen stream.

To determine the amount of thymol, we used high-performance liquid chromatography (HPLC) analysis. Chromatographic conditions were obtained using a Shimadzu model LC-20 instrument with a photodiode array (PDA) detector (Shimadzu, Japan). Acetonitrile and water (HPLC grade, Baker, ME, USA) were utilized as the organic mobile phase in a 50:50 ratio. The flow rate was 1 mL/min with an injection sample volume of 10 µL into a C18 column (Gemini-NX 5u, C18 110A, 250 × 4.6 mm, Phenomenex, CA, USA). The temperature was set at 20 °C, and the detection was monitored at a 275 nm wavelength. The unique peak of thymol was detected after 2.7 min. Thymol standard solutions in acetonitrile were prepared and used for calibration (Fig. S1).

### Thymol retention profile on porous metal

Thymol-loaded PLGA samples were prepared in advance and dried in a hood. Samples containing free thymol in acetonitrile were also prepared and dried in the hood to establish a reference control. The samples were left in the open air at room temperature and were provided by 5 random volunteers (20–30 years old) who smelled the sample every day and provided a score for odor intensity ranging from 0 to 10. For quantitative analysis of the thymol release, the samples were dissolved and sonicated in acetonitrile at different time points to determine the remaining amount of thymol using HPLC analysis using the same conditions as for content analysis of nanoparticles.

### Titanium 3D printing

Jewelry titanium objects were designed with AutoCAD® (version 2018.3, Autodesk, Inc., San Rafael, CA, USA), saved in their final form in STL format, and produced using an online 3D printing platform (i.materialise, Materialise, Belgium). The objects were printed using a titanium alloy called Ti-Al6-V4 by Select Laser Melting (SLM) technology. A high-powered laser selectively binds particles on the powder bed together while the machine distributes even layers of metallic powder. Support structures are automatically generated, built simultaneously in the same material, and later manually removed. Once complete, the part underwent heat treatment.

### Thymol retention in humidity conditions

Thymol-loaded PLGA nanoparticles were prepared and deposited on porous titanium as previously described. Electron deposition was used to add a 2 nm SiO_2_ layer using a base vacuum of about 10–6 Torr and a rate of 0.4 A/sec (8 kV e-beam evaporator, VST, Israel). Thymol content in samples was measured using HPLC, and samples were placed in lab-made humidity chamber for 3 days. The samples were then analyzed using HPLC to measure thymol residues.

## Results

### Aluminum porous structures

**Fig. 1:**
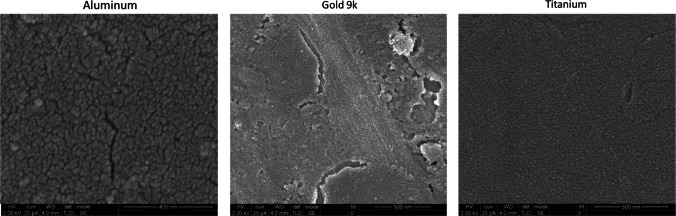
SEM characterization of the different metal surfaces. These images represent the surface structure of the different metals before etching treatments: aluminum, 9-karat gold alloy and titanium

**Fig. 2:**
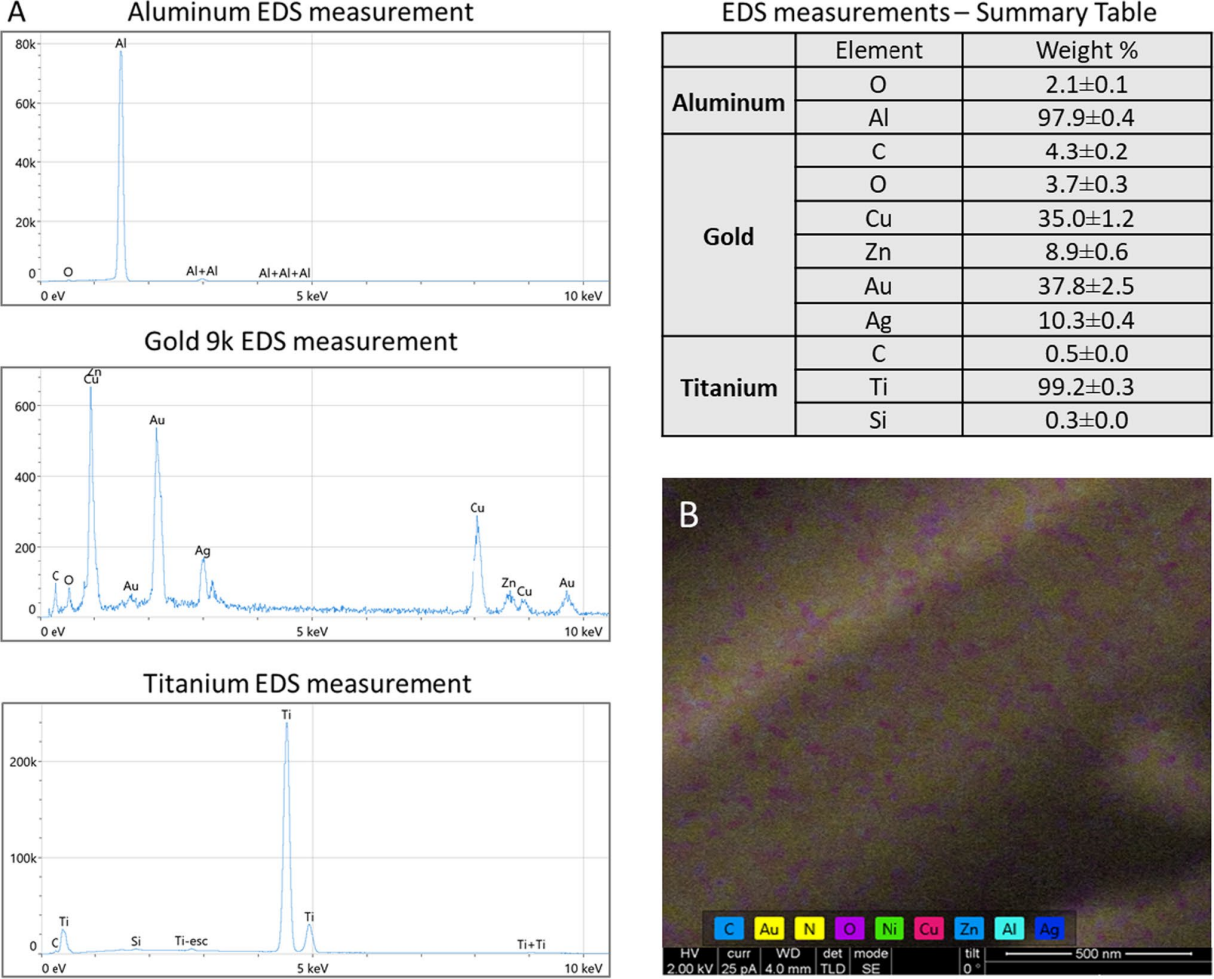
EDS characterization of the different metal surfaces. **A** EDS measurements showing the chemical element contents were realized for each metal sample: aluminum, 9-karat gold alloy, and titanium. Y-axis depicts the number of counts and the x-axis the energy of the X-rays. The position of the peaks leads to the identification of the elements and the peak height helps in the quantification of each element’s concentration in the sample. The aluminum sample contains 97.9% Al; the 9-karat gold alloy sample contains: 35% Cu, 8.9% Zn, 37.8% Au, and 10.3% Ag; and the titanium sample contains 99.2% Ti. **B** EDS measurement was directly realized on SEM image showing the different surface zones containing different chemical elements besides Au

**Fig. 3:**
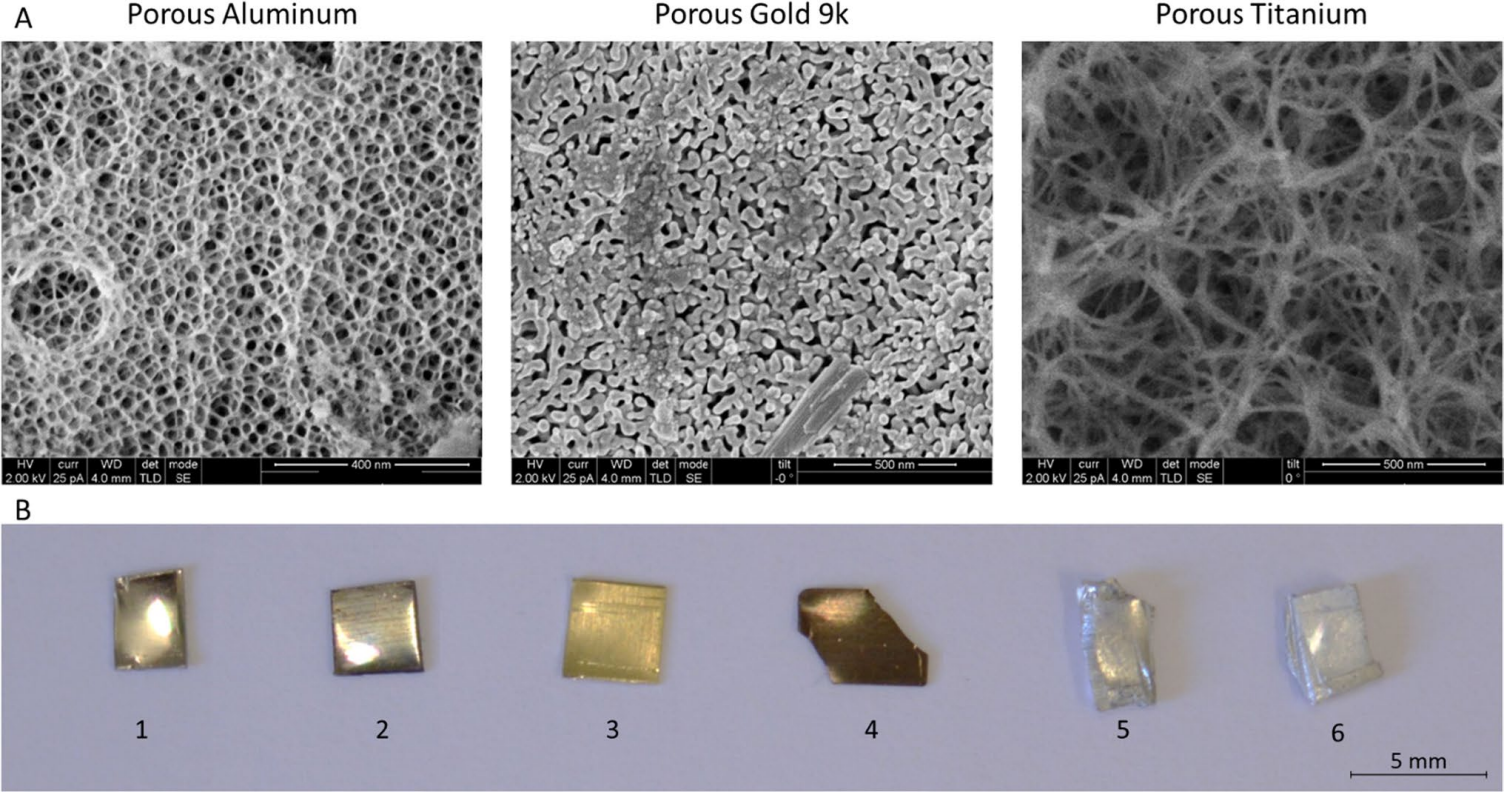
SEM characterization of the different porous metal surfaces. These images represent the nanostructures obtained in the optimal conditions of fabrication. **A** Aluminum nanostructures with about 30 nm pore size were obtained after dipping in HNO_3_ 35% for 35 min, gold nanostructures with about 30 nm pore size were obtained after dipping in HNO_3_ 35% for 72 h and titanium nanostructures with about 150 nm pore size were obtained after dipping titanium in NaOH 5 M for 24 h. **B** This picture represents the different metal pieces before and after the etching treatment: titanium (1. before, 2. after), 9-karat gold alloy (3. before, 4. after), aluminum (5. before, 6. after)

Aluminum reacts with nitric acid (HNO_3_) to form porous structures on the surface of the metal. Aluminum samples have a non-porous structure, as shown in SEM before HNO_3_ treatment (Fig. 1) and the EDS analysis confirmed that the samples were mainly composed of Al elements (Fig. 2A). Aluminum pieces were dipped in various HNO_3_ concentrations at different times (Table 1) to determine the ideal conditions to obtain porous structures. Only few porous structures were observed at short times and low concentrations (from 15 min at 15% and 30% to 30 min at 15%). However, the etching treatment was aggressive at longer times of 1, 4, and 24 h and at higher concentrations of 53% and 70%, destroying the porous structures. Only at the intermediate concentration of 35% during 30 min to 1 h of exposure, stable porous structures with a 30 nm diameter were produced (Fig. 3).

**Table 1:** Porous metals prospecting: table summary

	Solution	Time (h)	Concentration	Results
Aluminum	HNO_3_	0.25	15%	Few pores
			35%	Few pores
		0.5	15%	Few pores
			35%	~ 30 nm pores
			53%	No pores—aggressive treatment
			70%	No pores—aggressive treatment
		1	15%	Few pores
			35%	~ 30 nm pores
		4	15%	No pores—aggressive treatment
			35%	No pores—aggressive treatment
		24	15%	No pores—aggressive treatment
			35%	No pores—aggressive treatment
Gold 9 k	HNO3	0.5	35%	No pores—weak treatment
		24	15%	No pores—weak treatment
		24	35%	~ 30 nm pores
		48	15%	~ 15 nm pores
		48	35%	~ 30 nm pores
		72	15%	~ 30 nm pores
		72	35%	~ 30 nm pores
		96	35%	~ 15 nm—aggressive treatment
		121	35%	~ 15 nm—aggressive treatment
		146	35%	~ 15 nm—aggressive treatment
Titanium	NaOH	24	5 M	~ 150 nm pores
			7.5 M	No pores—aggressive treatment
			10 M	No pores—aggressive treatment
		48	5 M	~ 150 nm pores
			7.5 M	No pores—aggressive treatment
			10 M	No pores—aggressive treatment

### Gold porous structures

The gold alloy we used also has a smooth and non-porous structure, based on SEM measurements (Fig. 1). According to the EDS analysis, the gold alloy only comprises 37.8% Au; the remaining elements are 35% Cu, 8.9% Zn, and 10.3% Ag (Fig. 2A). Figure 2B presents the distribution of the chemical elements on the gold alloy surface before HNO_3_ etching. The non-gold chemical components were chemically etched away by reacting them with HNO_3_, leaving only pure gold. (Fig. 3). The optimal conditions for obtaining large nanopores were determined using different concentrations and soaking durations. At a short soaking time of 24 h, only a high concentration of 35% provided pores with a size of about 30 nm, whereas a lower concentration of 15% induced smaller (15 nm) or only sporadic pores. At longer soaking times of 72 h, 30 nm pores were obtained at both concentrations (15% and 35%). Longer soaking times of 96–146 h in HNO_3_ were too aggressive and not suitable for obtaining uniform pores (Table 1).

### Titanium porous structures

A series of different sodium hydroxide (NaOH) soaking solutions were prepared to determine the optimal conditions to produce titanium with large pores. SEM images of the titanium samples before chemical treatment showed a smooth structure (Fig. 1), and EDS measurements confirmed a content of 99.2% Ti elements (Fig. 2A). Only by using a 5 M NaOH solution for 24 h, make it possible to obtain porous structures ranging between 150 and 200 nm in diameter (Fig. 3). High concentrations of NaOH (7.5 and 10 M) were too aggressive and did not induce controllable porous structures. However, extending the duration of chemical treatments to 48 h had no impact on the enlargement of the porous structures (Table 1).

### Physicochemical characteristics of the metal surface

**Fig. 4:**
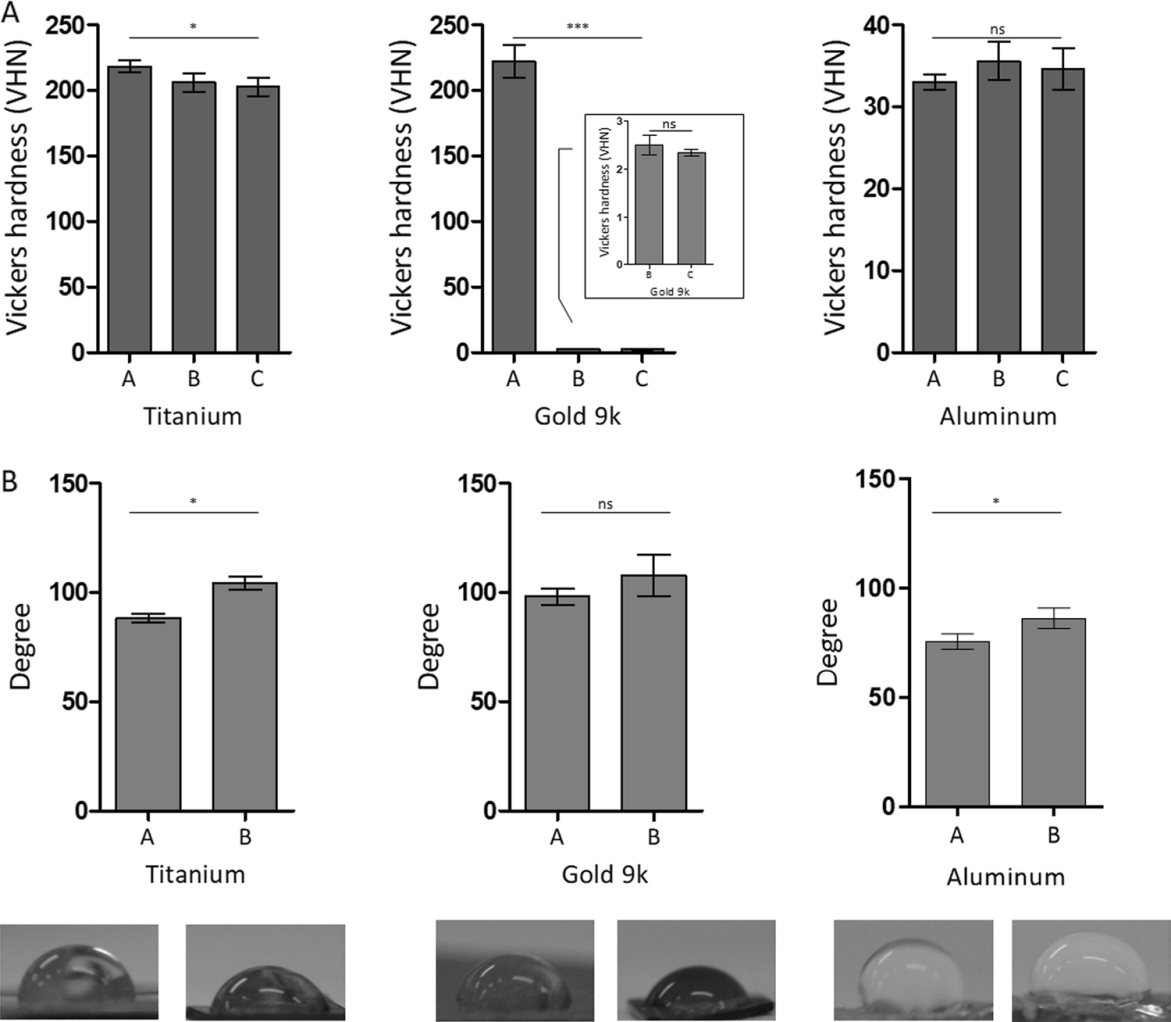
Physicochemical characteristics of the different metal surfaces. **A** Vickers hardness measurements performed on titanium, 9-karat gold alloy and aluminum before etching treatment [A], 24 h after [B] and 3 months after [C]. **B** Water contact angle measurements on titanium, 9-karat gold alloy and aluminum before etching treatment [A] and 24 h after [B]. Representative Images from each experiment is presented below the graphs. ns indicates no significant difference, n = 3, *p < 0.1, ***p < 0.001

To understand the long-term performance of the materials and the impact of the process on the samples, Vickers hardness measurements were performed before and after the etching treatment and 3 months post treatment. In the titanium surface, a slight decrease in the hardness properties was observed post etching, that remained unchanged over 3 months. In aluminum samples a neglectable mechanical increase was observed post etching. Nevertheless, a significant decrease in the hardness of the gold samples after the dealloying etching process was measured (Fig. 4A). Contact water angle measurements showed that the etching process improved the wettability of the samples (Fig. 4B). This result is correlated with the formation of pores on the surface of these metals.

### Thymol encapsulation in PLGA nanoparticles

**Fig. 5:**
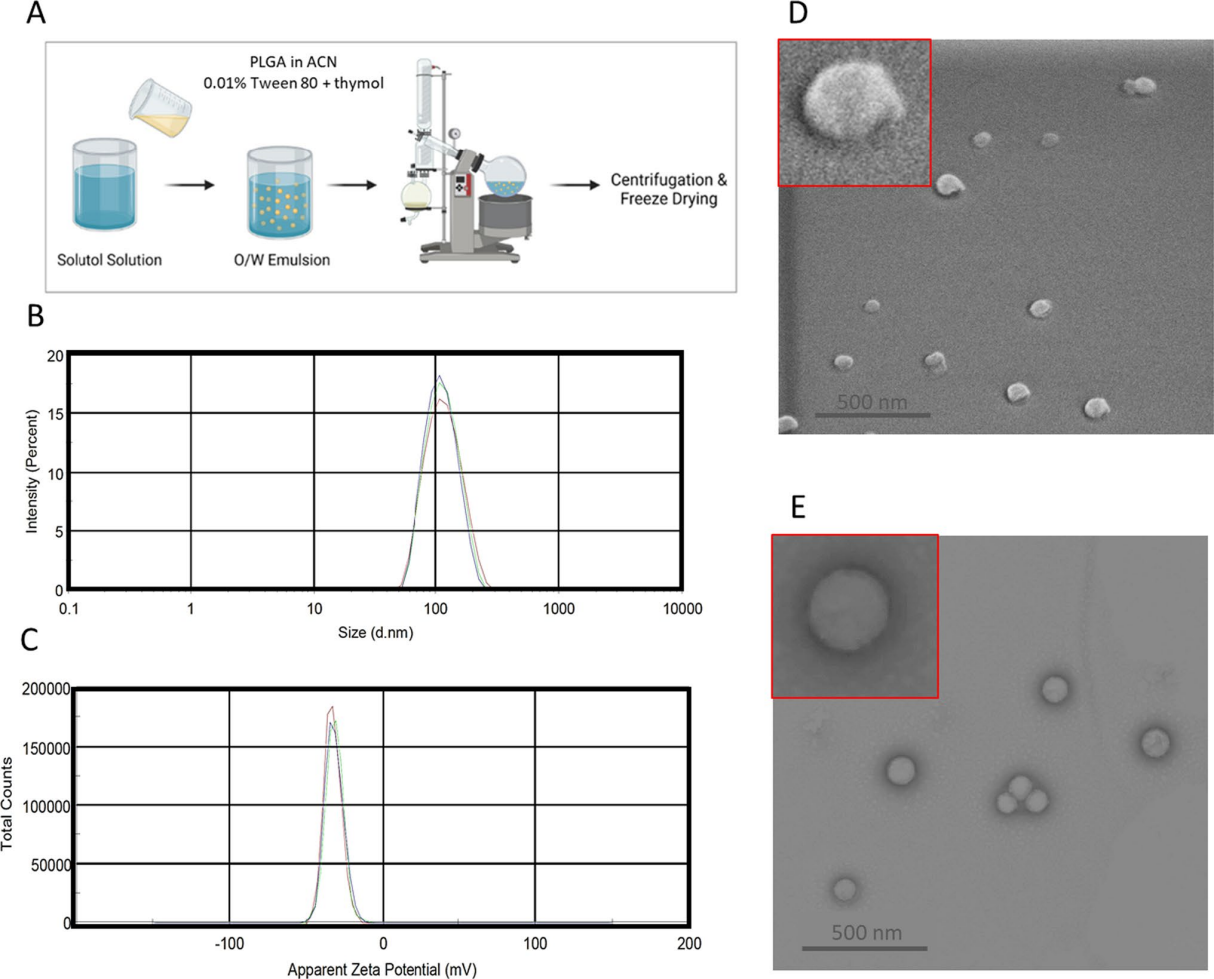
Thymol-loaded PLGA nanoparticle preparation and characterization. **A** Illustration of the encapsulation process of thymol into PLGA nanoparticles by emulsification-evaporation method (reproduced and adapted with authorization [41]). **B** DLS measurement of the nanoparticles **C** Zeta potential of the nanoparticles **D** SEM and **E** TEM Image of the nanoparticles

Thymol was encapsulated in PLGA nanoparticles using an emulsion solvent evaporation technique, shown in Fig. 5A. Thymol is a small hydrophobic molecule that can be entrapped in the matrix material during particle emulsification. The nanoparticle’s size was measured using the DLS method (165.6 nm ± 2.227; PDI = 0.079 ± 0.018) (Fig. 5B). The nanoparticles were negatively charged (− 32.1 mV ± 0.681) (Fig. 5C); the morphology of the particles was spherical with high uniformity (Figs. 5D, E).

### The release of thymol-loaded nanoparticles from the porous material

**Fig. 6:**
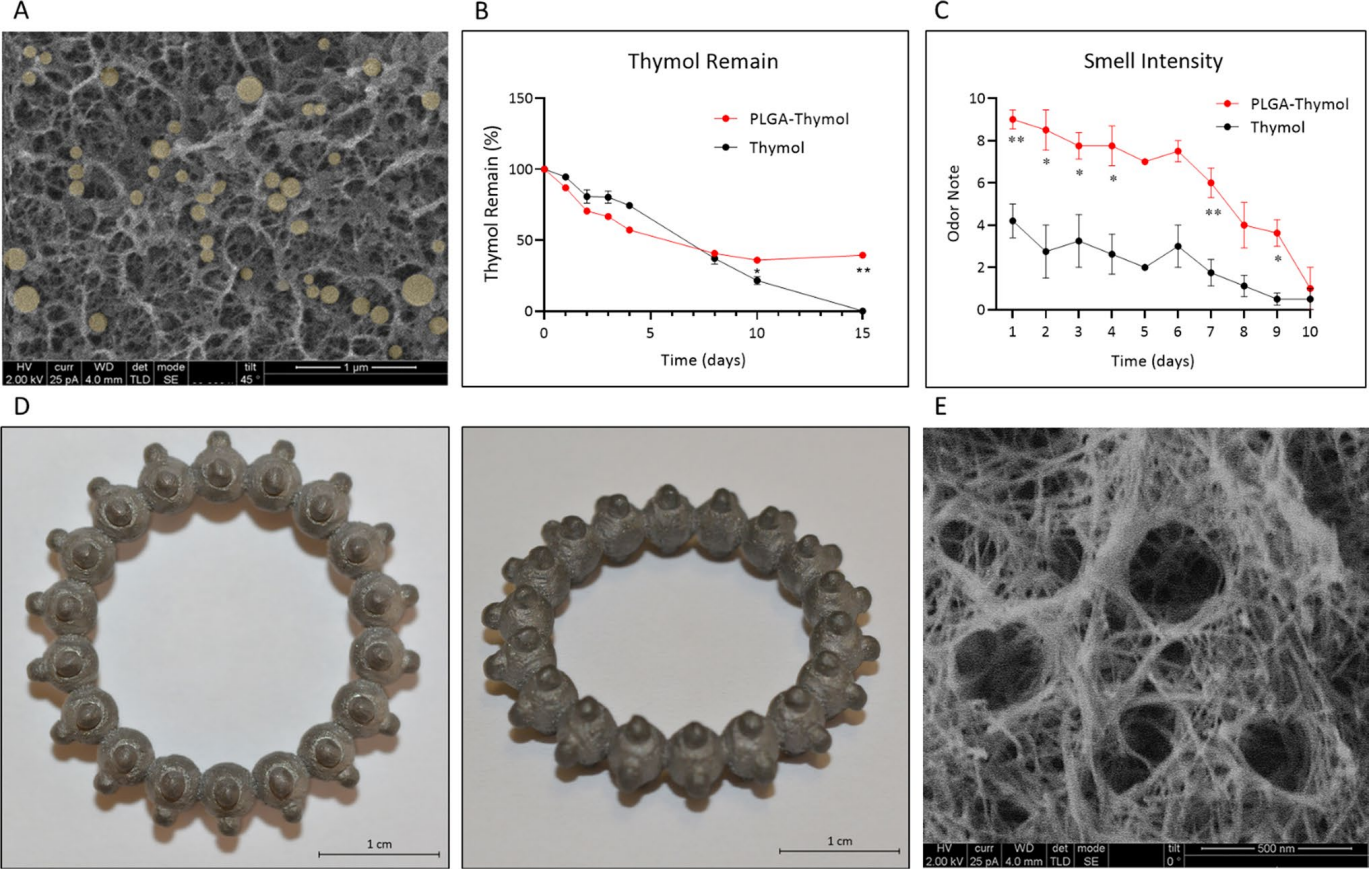
Thymol-loaded PLGA nanoparticles encapsulation in porous titanium structure. **A** SEM image of thymol-loaded PLGA nanoparticles in porous titanium (PLGA particles were post-highlighted in yellow). **B** Thymol release profile measurement using HPLC (from PLGA nanoparticles vs. free thymol). *p < 0.05, **p < 0.001. **C** Thymol release profile measurement using odor test (from PLGA nanoparticles vs. free thymol). *p < 0.05, **p < 0.001. **D** 3D printed titanium ring prototype before (left) and after etching treatment (right). **E** SEM image of the 3D printed titanium ring after etching treatment

The release profiles of thymol from the nanoparticle-loaded metals were compared with those of free thymol in open-air conditions. The nanoparticles were efficiently internalized into the porous titanium, with minimal residues on the outer surface, according to the SEM images (Fig. 6A). The release profile confirmed that after 15 days, the amount of thymol remaining in the samples was significantly higher than that of the free thymol. After 15 days, there was almost no thymol left in the free thymol samples, and there was nearly 40% of thymol when it was used in particles (Fig. 6B). Odor tests of the same samples for 10 days revealed that the samples containing thymol-loaded PLGA nanoparticles obtained higher scores for smell intensity than those that contained thymol, despite the identical initial quantities (Fig. 6C). Based on SEM, mPEG-b-PLA micelles (30 nm) were also deposited on porous aluminum, and silica nanoparticles (50 nm) were deposited on porous gold, confirming efficient incorporation into the metals (Fig. S2).

To provide a full prototype for smell-releasing jewelry, a titanium ring was designed and 3D printed. Figure 6D shows pictures of the titanium 3D-printed ring before and after treatment, showing no visual effect on the actual product. SEM images of the porous titanium printed ring were similar to the porous pure titanium structure (Fig. 6E). Such application may require further adjustments since jewelries may be exposed to the body and to humidity and, as a result, particles incorporated in the porous titanium may detach or degrade faster. Therefore, we explored the feasibility to fix and stabilize the particles using a thin fixation layer that may prevent hydrolysis and oxidation without affecting the apparency of the object. A thin layer of SiO_2_ (2 nm thick) was deposited on top of the metal surface pre-loaded with thymol PLGA nanoparticles. Figure S3A shows the differences in nanoparticle morphologies post exposure to high humidity for 3 days with or without a protective coating of SiO_2_. We found that despite the exposure to extreme humidity conditions, the particles coated with the SiO_2_ have maintained their structures, while in the absence of the protection, the particles were partially disintegrated. Moreover, as shown in Fig. S3B we demonstrate that the thin coating allows the release of the thymol with higher retention over 3 days in humidity, with 90% retention with the SiO_2_ layer and only 25% without the coating.

## Discussion

In this study, we described a versatile and cost-effective method for fabricating porous metals with controllable pore sizes. Metal dipping in etching solutions is a simple and inexpensive alternative process, thus holding significant promise for many applications for porous metals. The etching conditions were extensively calibrated, including testing different exposure durations, etching solution compositions, and varying concentrations of the solutions, leading to metals with controllable and relatively uniform pores. We found that porous morphology is dependent on the type of solvent and that the pore size and distribution are impacted by the etching solvent concentrations and the etching durations. High concentrations and long etching times typically result in larger pores, but they may also lead to non-uniform distributions or even complete dissolution of the metal as in the case of aluminum using a HNO_3_ concentration of 70% during 30 min or even using a HNO_3_ concentration of 15% during 4 h or 24 h. At the same time, lower concentrations and shorter etching times result in smaller pores or even the absence of enough etching influence as can be shown in the case of aluminum using a HNO_3_ concentration of 15% during 30 min or with 35% during 15 min. In the other materials similar adjustments made it possible to control the pore size of the final structure. According to our needs, we successfully created surfaces with pores of dozens of nanometers, which could be used for particle entrapment. In the future, some improvements, based on combining several solvents [18] and integrating computational studies [19, 20] could be made to gain even finer control or to obtain larger pores to allow the incorporation of micron-sized particles using this method.

To provide a wider demonstration, we used different metals: aluminum, gold, and titanium as substrates. Etching processes in aluminum were previously described for different acid solvents; however, obtaining nanoporous structures remains a challenge, since most large micropores were obtained using hydrochloric acid (HCl) [21, 22], or even using oxalic acid/phosphoric acid mixtures (C_2_H_2_O_4_/H_3_PO_4_) [23]. On the other hand, nanopores were obtained using an anodizing process involving sulfuric acid (H_2_SO_4_) dipping [24]; however, the small dimensions of the pores prevented the further deposition of nanoparticles. In our case, when we used HNO_3_ (35% during 35 min) as the etching chemical to obtain nanopores via a simple dipping process, a homogeneous structure with pore sizes of ~ 30 nm was formed enabling the deposition of mPEG-b-PLA micelles.

With gold surfaces, we used HNO_3_ because of its common use as a method for determining the purity (karat) of gold by breaking down other surrounding chemical elements [25]. This dealloying method is based on the observation that HNO_3_ does not affect the gold elements. To date, it has been shown that this approach could produce tiny pores in a gold alloy, which range in size from 2 to 5 nm [26, 27]. Therefore, to create bigger pores, we used a 9-karat gold alloy with high quantities of non-gold chemical elements to take advantage of this principle and to produce rather wide pores of about 30 nm (using HNO_3_ 35% for 72 h) enabling the deposition of silica nanoparticles. Further reducing the alloy purity could enhance the pore mesh size.

Titanium, unlike gold and aluminum, does not react with acid; therefore, NaOH is typically used to induce porosity [28, 29]. Our interest in titanium is beyond the scope of this study since this material is widely used in implantable devices and thus potentially benefits from our approach [30, 31]. Different NaOH concentrations and dipping conditions were used to optimize the production of uniformly porous titanium with controllable pore size and larger pores, which were ideal for incorporating slow-releasing particles. Finally, the ideal pore size was obtained by dipping the titanium samples in NaOH 5 M for 24 h.

Vickers mechanical strength measurements have shown that the metal surface hardness is maintained throughout the process and that this property is conserved even after several months. In the case of aluminum, a non-significant increase in the hardness properties was observed after the etching process. Kim et al. also described such increase in mechanical strength of porous aluminum according to the intensity of the production process [32]. In titanium, a slight decrease in the Vickers hardness values was observed. Similar results were shown by Furumoto et al. where significant decrease of Vickers hardness where observed depending on the increasing porosity of titanium [33]. In our case, the smaller modifications of the hardness properties in both aluminum and titanium can be explained by the utilization of a less aggressive treatment. In the case of gold, unlike the other metals, a significant decrease in mechanical strength was observed post etching. Gold, unlike aluminum and titanium, has undergone a dealloying process. According to the EDS measurements, only the Au element was left in the surface after the gold treatment, making it softer than before the etching process where the material contained chemical elements that contributed to the hardness. In a similar way, Li et al. [34] found that pure gold has a significant lower Vickers hardness value than gold alloys. These results also confirmed high stability and mechanical durability of the porous materials over time even several months after their production. Moreover, the contact angle measurements allowed to assess an increase in the wettability of the samples after the etching process. This increase can be directly related to the formation of porous structures at the surface of the metal samples as previously shown [35].

In our mechanical deposition approach, nanoparticles were designed and fabricated to fit the pore size on the metal to allow proper entrapment. The high surface area of porous materials also improved the loading capacity of the metal to absorb the nanoparticles. As a demonstration for our method, thymol odor molecules were encapsulated in polymeric nanoparticles. The challenge of encapsulating odor molecules has been known in everyday consumables such as cleaning products, pesticides, perfumes, textiles, toiletries and more. In these regards, the insertion of nanoparticles in porous structure of the metals, by itself, has an advantage compared to other methods of deposition. Indeed, the incorporation of nanoparticles does not affect the external appearance of the metal which is a considerable important when aesthetic parameters are accounted for. 

Blind odor tests performed by volunteers revealed an efficient smell perception lasting 10 days in metals incorporated with thymol-loaded PLGA nanoparticles. Moreover, it was found that the intensity of the thymol smell was higher in metals containing PLGA nanoparticles in comparison with the free thymol-covered metal surfaces, despite the equal total quantities of thymol that were deposited in each sample surface. These results show that the PLGA nanoparticles enhance the sense of smell over a longer time. This result requires further confirmation, and it may offer future research perspectives in the field of odor perception.

Release studies of thymol from the nanoparticles deposited in porous titanium showed a chemical retention of almost 40% after 15 days and odor retention for 10 days. This is possibly due to the special properties of the encapsulation material, PLGA, which is a biodegradable polymer that decomposes when exposed to water. In our case, because the product is dry, it is probable that the degradation is negligible for many weeks and that it is possibly slightly affected by the humidity of the air, and that the main mechanism to release the entrapped compound is controlled by diffusion through the polymer chain network. Similar attempts to enhance smell retention have shown 40% volatile retention after 10 days from chitosan nanoparticles [36] and 50% after 15 days from gelatin–Arabic gum [37]. However, encapsulating volatile molecules on dry surfaces for longer periods remains challenging. Odor release depends on several parameters, such as the air/liquid interface release, the encapsulation technique, the material type, and the odor molecule used [15, 38]. Unfortunately, few studies have compared different release conditions to identify the specific parameters required to improve retention [39].

The blind odor tests and the HPLC measurements correspond to the preliminary studies required in the cosmetic and perfume industry to assess the release kinetics of different odor formulations. In the future, additional analytical methods should be considered, such as Gas Chromatography-Mass Spectrometry (GC–MS) and Solid-Phase Microextraction (SPME), to measure the concentration of odor molecules released in the air over time. Furthermore, safety and regulatory tests should be performed to assess that the technology does not cause any toxic effects in contact with the body [40].

Finally, a titanium ring was created using advanced metal 3D printing techniques. The external visualization of the object was unaffected by the etching and particle deposition processes. This jewel prototype could be potentially utilized as a portable system for porous materials that release odors. In these applications, the device might be subjected to humid environment that could lead to an accelerated degradation of particles in the porous titanium. Therefore, we explored the feasibility of using very thin layer of external material to increase stability. A thin layer of SiO_2_ (2 nm thick) was applied to the metal surface that had previously been coated with thymol-loaded PLGA nanoparticles to avoid hydrolysis. SiO_2_ was selected according to its hydrophobic properties [41]. The deposition of SiO_2_ was made using e-beam evaporation method, using vertical deposition. This technique avoids a complete blockage of the release from the particles by remaining some of the particle surface uncovered. As a result of the protective layer, when exposed to humidity, the particles covered with SiO_2_ preserved their morphologies and improved the thymol retention without completely preventing release. This concept should be further studied using various layer thickness and various materials. The described prototype provides a proof of concept rather than a commercially-ready technology and can serve as a starting point for future product and for additional similar odor-releasing objects or devices. Titanium is also frequently employed in other fields such as the biomedical sector; therefore, this innovation, based on nanoparticles released from porous structures, should be considered in this field as well.

## Conclusion

In the presented work, different metals were used to produce porous surfaces, avoiding over-demanding methods. Different calibrations using etching solvents were carried out, producing sufficiently large nanoporous structures. By departing from the current approach to produce metal-eluting platforms, a major advantage emerged, since nanoporous structures could entrap nanoparticles in them. These nanoparticles contained a volatile compound, thus enabling retention and providing a better odor release. This work paves a new way to produce eluting metals by applying a new method of porous metal fabrication and slow-release formulations.

## Supplementary Information

**Additional file 1.** Supplementray figures.

## Data Availability

The datasets generated during and analysed during the current study are available from the corresponding author on reasonable request.
