# Supplementary material for: Generating porous metal surfaces as a mean to incorporate thymol-loaded nanoparticles
Source: Discov Nano. 2023 Jun 20;18(1):89. doi: 10.1186/s11671-023-03854-0 (PMC10281935; doi:10.1186/s11671-023-03854-0)
Supplement: Supplementary file 1 — Additional file 1. Supplementray figures. [file 11671_2023_3854_MOESM1_ESM.docx]

Supplementary Information

**Generating porous metal surfaces as a mean to incorporate thymol-loaded nanoparticles**

Chalom Zemmour^1^, Sofya Zakharova^2^, and Ofra Benny^1^

^1^ Institute for Drug Research, School of Pharmacy, Faculty of Medicine, The Hebrew University of Jerusalem, Jerusalem, Israel, 91120, [ofra.benny@mail.huji.ac.il](mailto:ofra.benny@mail.huji.ac.il) (corresponding author)

**^2^ Bezalel Academy of Arts and Design Jerusalem, Jerusalem, Israel**


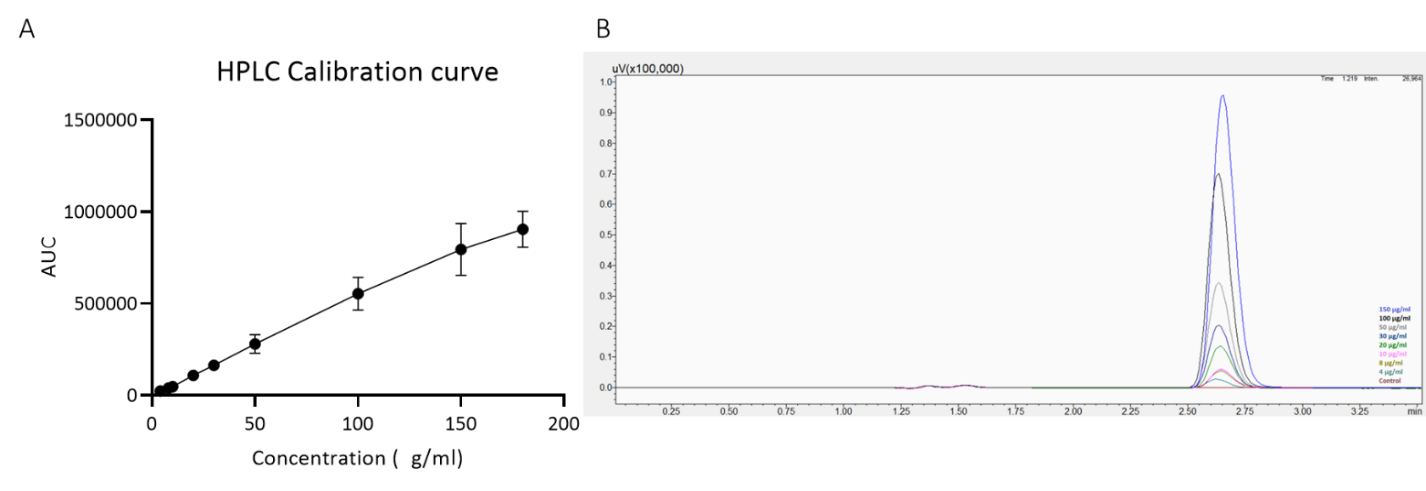


**Figure S1. Thymol HPLC calibration. (A)** HPLC calibration curve using thymol samples from 4µg/ml to 180µg/ml (calibration linear factor R^2^= 0.9972). **(B)** HPLC peaks of thymol in acetonitrile after 2.65 min. The peaks were detected at 276nm.


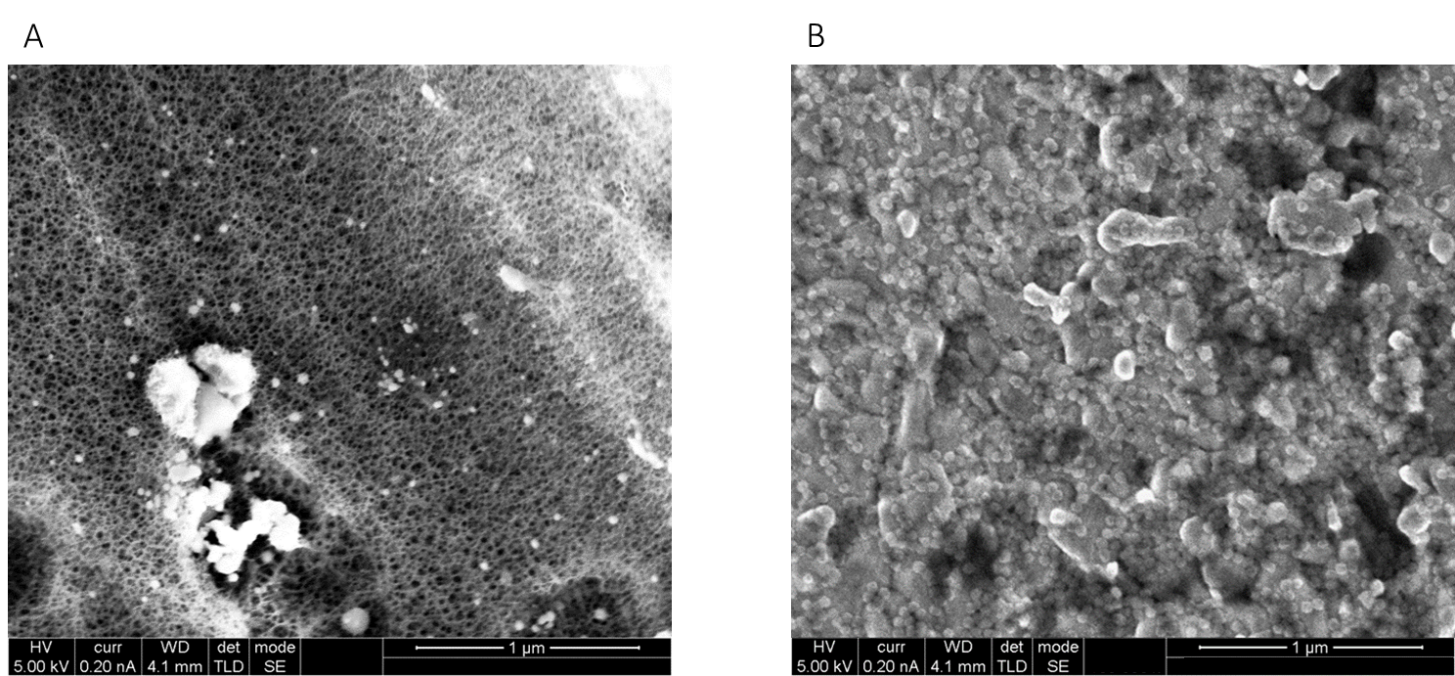


**Figure S2. SEM characterization of nanoparticles entrapped in aluminum and gold porous metal surfaces. (A)** mPEG-b-PLA nanoparticles entrapped in porous aluminum**. (B)** Silica nanoparticles entrapped in porous gold.


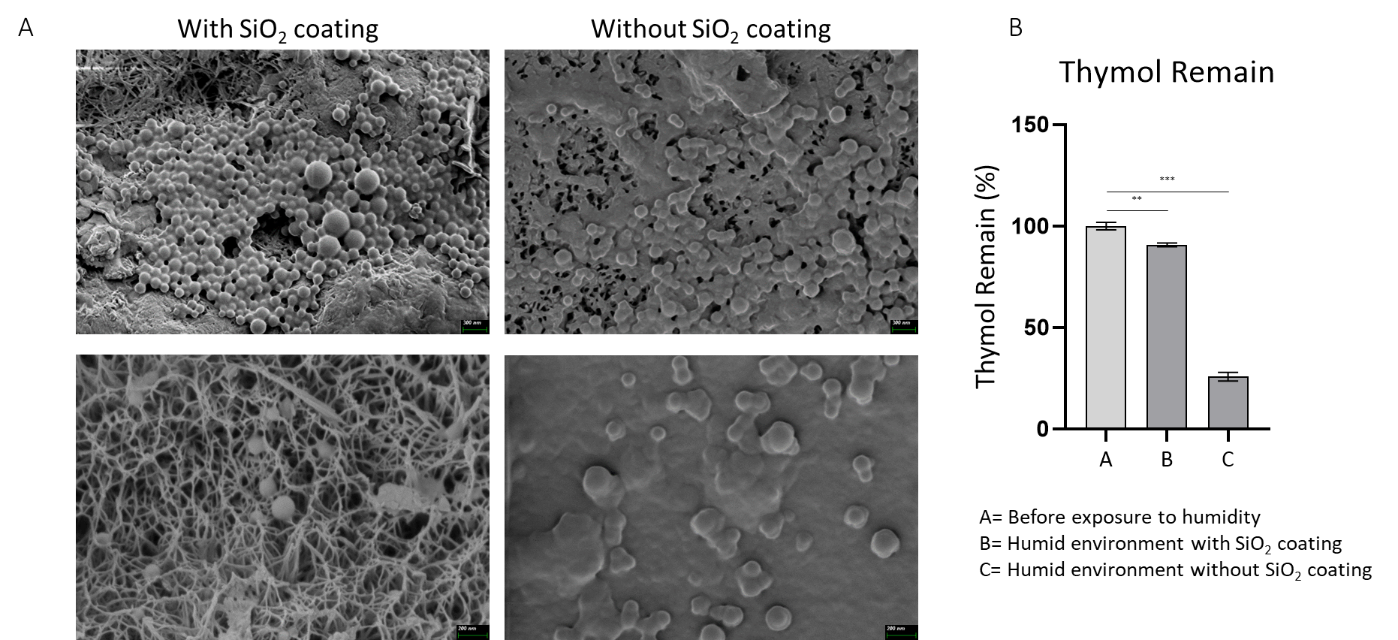


**Figure S3. Characterization and release profile of thymol-loaded PLGA nanoparticles in porous titanium after 3 days in a humidity chamber. (A)** SEM image of thymol-loaded PLGA nanoparticles in porous titanium after 3 days in a humidity chamber. The left images represent the nanoparticles covered with a thin layer of SiO_2_. The right images represent the nanoparticles that did not undergo SiO_2_ deposition. **(B)** Thymol release profile measurement using HPLC: [A] before humidity exposure, [B] with SiO_2_ deposition and after 3 days in a humidity chamber, [C] without SiO_2_ deposition and after 3 days in a humidity chamber (from PLGA nanoparticles vs. free thymol). ** *p* < 0.005, *** *p* < 0.001
